# Burst Pressure and Fatigue Durability of Commercially Available Duraplasty Sealants

**DOI:** 10.1002/alr.70132

**Published:** 2026-03-13

**Authors:** Katherine L. Lauritsen, Aishwarya V. Menon, Kaete A. Archer, Myah D. Webb, Jonathan Y. Ting, Jonathan J. Wilker, Julie C. Liu, Vijay R. Ramakrishnan

**Affiliations:** ^1^ Department of Otolaryngology‐Head and Neck Surgery Indiana University School of Medicine Indianapolis Indiana USA; ^2^ Rutgers New Jersey Medical School Newark New Jersey USA; ^3^ Davidson School of Chemical Engineering Purdue University West Lafayette Indiana USA; ^4^ Department of Chemistry Purdue University West Lafayette Indiana USA; ^5^ School of Materials Engineering Purdue University West Lafayette Indiana USA; ^6^ Weldon School of Biomedical Engineering Purdue University West Lafayette Indiana USA

**Keywords:** CSF Rhinorrhea, Endoscopic Skull Base Surgery, Intracranial, dura, Sinus Surgery, Skull Base, Skull Base Repair

## 1 Introduction

Methods of dural repair are critical for the prevention of cerebrospinal fluid (CSF) leaks in endoscopic skull base surgery (ESBS) [1]. The use of tissue sealants in ESBS was supported by early studies using porcine models showing a reduction in CSF leaks [2]. Surgeons have employed several methods of use, including overlaying sealants to act as watertight seals, adhesive layers between a multilayer repair, and as fillers to minimize dead space within the repair [1]. The American Rhinologic Society emphasizes that there is limited evidence supporting tissue sealant effectiveness [3]. Despite the lack of empirical data, aggregate dural sealant use occurred in over 50% of the dural defect repairs in studies from a recent systematic review [1].

**Key Points**

- Compare burst pressure and cyclic fatigue of sealants in an in vitro porcine dural repair model.
- Large variability observed in the performance of commercially available tissue sealants.
- Sealant selection and application are important considerations to achieve the desired function.

Both fibrin‐based sealants, typically sourced from animals or humans [1] and including the hemostatic components thrombin and fibrinogen, and polyethylene glycol (PEG)‐based sealants, composed of lab‐designed bioinert compounds [4], are used by surgeons in ESBS. In this study, the fibrin‐based sealants used were TachoSil, TISSEEL, and VISTASEAL, and PEG‐based sealants included DuraSeal, Adherus, and Coseal.

Existing literature examining dural sealants relies on simple burst pressure models that may not capture rapid or cyclic intracranial pressure fluctuations. Surgical utility in the immediate postoperative setting, when intracranial pressures may be volatile, and graft adherence is uncertain, remains undefined. Experimental studies that compare current commercially available sealants remain limited. To address these gaps, this in vitro study examines burst pressure at clinically relevant time points, and cyclic testing evaluates repair durability.

## 2 Methods

We studied six different commercial sealants using burst pressure and cyclic pressure testing at multiple time points in a 1 cm defect model. Detailed methods can be found online in the Supporting Information 1. A supplementary (Figure S1) and (Video S1) of the laboratory setup can also be found online in the Supporting Information folder.

## 3 Results

**FIGURE 1:**
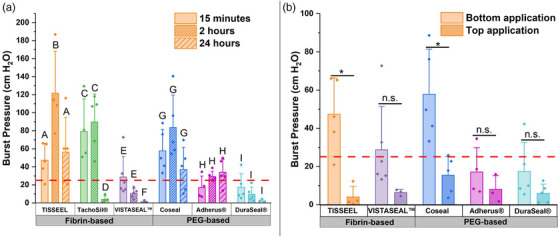
(a) Burst pressures of six sealants were tested at cure times of 15 min, 2 h, and 24 h, in a 1 cm defect model. The dotted line shows the high normal intracranial pressure value (25 cm H_2_O) [8]. Different letters indicate distinct Tukey groups within each sealant (*p* < 0.05). A Box‐Cox transformation was applied to the VISTASEAL data due to non‐normal data distribution. Data shown as mean ± standard deviation. (b) The sealants were cured for 15 min, and then, to mimic current intraoperative usage, the sealants were tested when placed above the graft. The dotted line shows the normal intracranial pressure value (25 cm H_2_O) [8]. *indicates statistical significance (*p* < 0.05), whereas n.s. indicates no statistical significance (*p* > 0.05). Data shown as mean ± standard deviation.

The burst pressure strength of all fibrin sealants except TISSEEL significantly decreased over time, whereas those of PEG‐based sealants were unaffected by cure time (Figure 1a and Table S1). Burst pressure was significantly higher for TISSEEL at 2 h than at 15 min (*p* = 0.02) and 24 h (*p* = 0.04). Burst pressures for TachoSil and VISTASEAL were significantly lower at 24 h than at 15 min (*p* = 0.008 for TachoSil and *p* = 0.0002 for VISTASEAL) and 2 h (*p* = 0.002 for TachoSil and *p* = 0.006 for VISTASEAL).

The average burst pressure for TISSEEL and Coseal significantly decreased when tested using the top sealant application technique compared to when the sealant was applied beneath the graft (*p* = 0.003 for TISSEEL and *p* = 0.006 for Coseal) (Figure 1b). The bottom sealant application technique also yielded higher average burst pressure values for all other sealants, although there were no statistically significant differences.

**FIGURE 2:**
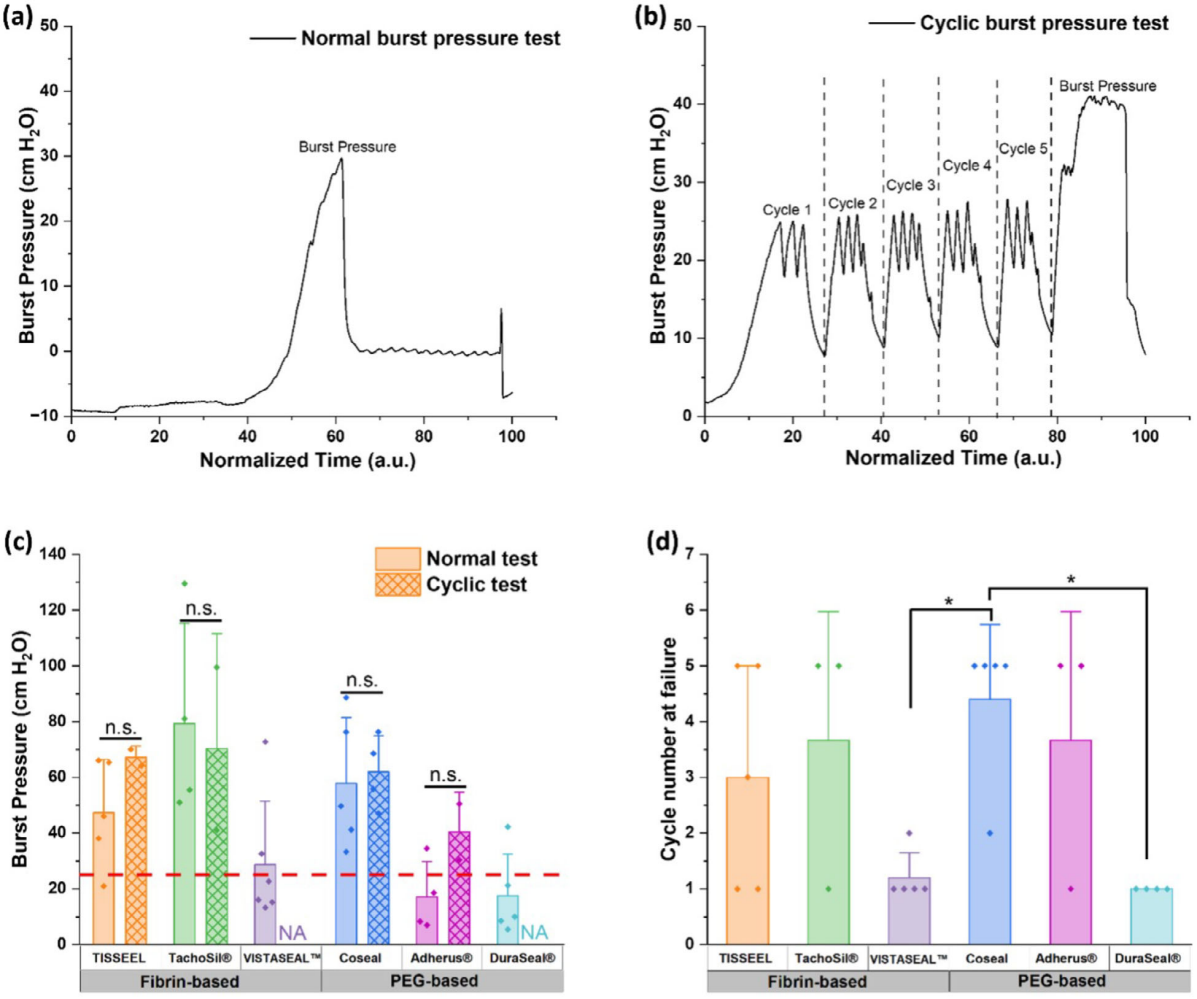
Aside from VISTASEAL and DuraSeal, the terminal burst pressures of all other sealants were unaffected by the cyclic fatigue. Figure 2 shows typical burst pressure versus time curves for (a) simple burst pressure test and (b) cyclic burst pressure test with five cycles of 15 s each. (c) A comparison of burst pressures obtained from single burst pressure tests with those obtained after cyclic burst pressure tests. Data for the normal burst pressure tests were re‐graphed from Figure 1a. The dotted line shows the normal intracranial pressure value (25 cm H_2_O) [8]. Due to non‐normal data distribution, a non‐parametric Wilcoxon test with *α* = 0.05 was used to test statistical significance. (d) Average cycles sustained by each sealant during the cyclic test. TISSEEL and TachoSil were able to withstand 3 and 4.6 cycles on average, respectively. Coseal and Adherus withstood 4.9 and 4.5 cycles, respectively. A non‐parametric Wilcoxon test with *α* = 0.05 was used to test statistical significance. The sealants were applied using the bottom application method and cured for 15 min. *Indicates statistical significance (*p* < 0.05), whereas n.s. indicates no statistical significance (*p* > 0.05). NA = Not Applicable, as the sealants were unable to withstand cyclic pressure testing.

All sealants except VISTASEAL and DuraSeal withstood cyclic pressure application (Figure 2d). The average burst pressure for all other sealants, after cyclic pressure application, was not significantly altered compared to the average single burst pressure (Figure 2c).

## 4 Discussion

Our study design aimed to simulate real‐life clinical scenarios. Given that sealants may be the final step in repair, an early time point can reflect emergence from anesthesia and postoperative stressors. After 2 h, patients may engage in eating, drinking, speaking, sitting up, or experience episodes of nausea/emesis. Finally, the assessment at 24 h after surgery may reflect diurnal pressure fluctuations, when the repair may be most vulnerable.

Most sealants withstood high‐normal intracranial pressures, with the exception being DuraSeal. Two fibrin‐based sealants, TachoSil and VISTASEAL, showed significant declines in performance over 24 h, whereas TISSEEL maintained good performance and peaked at 2 h, possibly due to its compact tissue interface seen in prior histological studies compared to a porous TachoSil [5]. TISSEEL's endurance suggests suitability for patients at risk for early postoperative stress in addition to hemostatic benefits. PEG‐based sealants were unaffected by cure times and exhibited consistent performance. Understanding the role of reabsorption and maturation at the repair site may elucidate the differences in sealant performance over time.

Our findings demonstrate that sealant placement between the dural‐graft interface improved the burst pressure for all sealants, with significance in TISSEEL and Coseal. This finding contrasts with a prior in vitro study [6]. Important differences that may account for this variability are the larger defect size for our study (1 cm vs. 3 mm) and the inclusion of the graft layer [6]. Applying the sealant using the top application technique allows the sealant to act as a surface protectant for structural support. The bottom application technique suggests that the dural sealants may also have utility as an adhesive interface, providing a stronger mechanical hold. Physicians’ hesitance with placing sealants beneath the graft is largely due to concerns that it will interrupt the dural‐graft interface and/or swell, which could potentially impair healing [7]. Further studies can assess outcomes of sealant placement and techniques, as well as explore their utilization using in vivo animal models.

Cyclic burst pressure testing was performed to investigate the effect of sealant fatigue from normal intracranial pressure fluctuations related to daily function. VISTASEAL and DuraSeal were unable to withstand cyclic testing, an effect that is not unique to either of the two different sealant families. These findings can aid in clinical decisions and enhance confidence in their reliability. While we cannot account for patient‐specific differences, such as their innate healing response, these data offer specific insights to refine surgeons’ sealant selection and use. Further directions may include randomized‐controlled in vivo trials to continue to examine various surgeon closure techniques with sealants and enhancements in dural sealant formulations that result in the next generation of biomaterials.

## Funding

This project was funded by the Engineering in Medicine Initiative, a partnership between Indiana University School of Medicine and the Purdue University College of Engineering (J.C.L, J.Y.T, and V.R.R.). Cook Biotech Incorporated (d/b/a Evergen) donated the Biodesign Duraplasty Graft, and Johnson & Johnson MedTech donated the VISTASEAL fibrin sealant used for all experiments. The authors also acknowledge funding from the National Science Foundation (grant DMR‐2104783 to J.C.L. and J.J.W.), Davidson School of Chemical Engineering, Purdue University (J.C.L. and A.V.M.), Department of Chemistry, Purdue University (J.J.W. and A.V.M.), and a Lillian Gilbreth Postdoctoral Fellowship from Purdue University College of Engineering (A.V.M.).

## Conflicts of Interest

The authors declare no conflicts of interest.

## Supporting information




**Supporting File 1**: Commercial FDA‐approved surgical sealants were studied in combination with a graft (Biodesign Duraplasty Graft) to test their potential to seal dural leaks in minimally invasive endoscopic skull base surgeries. **(a)** Schematic showing two different sealant applications studied here. Sealant was either applied between the Biodesign graft and dura in the “bottom application” or above the Biodesign graft in the “top application”. **(b)** Set up for burst pressure test.

**Supporting File 2**: alr70132‐sup‐0002‐Figure.pdf

**Supporting File 3**:alr70132‐sup‐0003‐VideoLabels.mp4
