# Supplementary material for: Burst Pressure and Fatigue Durability of Commercially Available Duraplasty Sealants
Source: Int Forum Allergy Rhinol. 2026 Mar 13;16(4):418–21. doi: 10.1002/alr.70132 (PMC13047941; doi:10.1002/alr.70132)
Supplement: Supplementary file 1 — Supporting File 1: Commercial FDA‐approved surgical sealants were studied in combination with a graft (Biodesign Duraplasty Graft) to test their potential to seal dural leaks in minimally invasive endoscopic skull base surgeries. (a) Schematic showing two different sealant applications studied here. Sealant was either applied between the Biodesign graft and dura in the “bottom application” or above the Biodesign graft in the “top application”. (b) Set up for burst pressure test. [file ALR-16-418-s002.docx]

**Materials:**

Porcine dura mater was obtained from Animal Technologies, Inc., and Biodesign^®^ Duraplasty Graft was obtained from Cook Biotech Incorporate (d/b/a Evergen).^15^ The size of the Biodesign^®^ graft used for all studies was 2.0 cm x 2.5 cm x .25 mm. We tested several widely used dural sealants commercially available today. The commercial FDA-approved surgical sealants TachoSil, TISSEEL, DuraSeal, Adherus, and Coseal were purchased from synergysurgical.com. VISTASEAL was obtained from Johnson & Johnson MedTech.

**Methods:**

*Burst Pressure Testing:*

Porcine dura was stored at -20 °C. It was thawed at 4 °C overnight and hydrated in phosphate-buffered saline (PBS) before use. The dura was patted dry and placed exterior side up, and a biopsy punch was used to make a 1 cm puncture on the left or right hemisphere of the dura.

To test the effect of sealant application location relative to the graft, we first applied sealant beneath the graft (“bottom application”). Next, 150 µL of combined sealant was applied around the 1 cm defect, and then the Biodesign graft was pressed on top to ensure a leakproof seal (**Supplemental** **Figure 1a**). To mimic current intraoperative conditions and a common endoscopic application technique, we subsequently performed identical experiments where the Biodesign^®^ graft was placed over the dural puncture as an onlay, and then the sealant was placed over the graft and surrounding dura (“top application”). The application of Biodesign was omitted in the case of TachoSil. A TachoSil patch was cut into 2.0 cm x 2.5 cm sections and pressed directly onto the puncture for approximately 30 seconds.

Sealants were applied and then cured for 15 minutes, 2 hours, or 24 hours. The 15-minute cure was performed at room temperature, and for 2- and 24-hour cures, the repairs were maintained in a humidified incubator at 37 °C and equilibrated at room temperature prior to testing. Tissues were covered with wet paper towels and sealed with a layer of cling wrap to prevent desiccation during the 24-hour time period. For the experiments using the “top application”, only the 15-minute cure time was tested.

Burst pressures were obtained using the American Society for Testing and Materials (ASTM) standard F2392-04 for burst strength of surgical sealants^16^ with some modifications to the test protocol (**Supplemental Figure 1b**). Specifically, a custom-made burst pressure apparatus was designed to accommodate a 1-cm defect size. Water dyed with red food coloring was used as the test fluid and was pumped into the apparatus at a rate of 500 mL/h. The burst pressure tests were designed to mimic a typical overlay (onlay) graft placement, and thus, the water column was always in contact with the dura.^17^ After activating the device, a syringe pump was mechanically compressed to displace water towards the apparatus holding the dura/graft/sealant. As water was displaced towards the enclosed chamber, pressure was applied to the dural defect until a burst pressure was met. Pressure was monitored using the Omega Digital Transducer Application, which has a real-time graphical display showing the gradual increase in pressure until a rapid drop-off was achieved that signaled the burst pressure of the dura/graft/sealant. Concurrently, repairs were visualized for leakage of dyed fluid and carefully examined for signs of dura/graft damage or graft/sealant dehiscence.

*Cyclic Fatigue Testing:*

The same apparatus was used for cyclic burst pressure testing. Samples were prepared as described previously and cured for 15 minutes before testing. Once the samples were mounted on the burst pressure apparatus, the syringe pump was activated, and the pressure was manually cycled between 20-25 cm H_2_O over a 15-second interval. After this interval, the syringe pump was turned off for 15 seconds, during which the sample was inspected for leaks. If no leak was observed, the cyclic testing process was repeated until a leak was detected or for a total of 5 cycles, whichever occurred first. If all 5 cycles were completed without leakage, the syringe pump was reactivated until burst pressure was reached to evaluate the effects of fatigue on burst pressure performance.

*Statistical Analysis:*

Statistical analysis was performed using JMP software. At least 5 replicates were tested in all cases. When comparing two groups, a two-tailed Student’s t-test or Dunnett’s test with equal variances was used with α = 0.05. When comparing more than two groups, analysis of variance (ANOVA) was used with α = 0.05. Normality was confirmed using the Shapiro-Wilk test, and equality of variance was validated using the Brown-Forsythe test. In the case of non-normal residuals or unequal variances, an appropriate transformation (e.g., Box-Cox transformation) was applied to the data to facilitate statistical comparisons. A Tukey posthoc test was used to determine statistically significant groups. Statistical significance is indicated by letters such that groups that do not share the same letter are statistically different (p < 0.05). If no successful transformations were obtained, a non-parametric test (Wilcoxon test with α=.05) was used to determine statistical significance.
