# Supplementary figures and images for: Burst Pressure and Fatigue Durability of Commercially Available Duraplasty Sealants
Source: Int Forum Allergy Rhinol. 2026 Mar 13;16(4):418–21. doi: 10.1002/alr.70132 (PMC13047941; doi:10.1002/alr.70132)

(a)

**Bottom application**

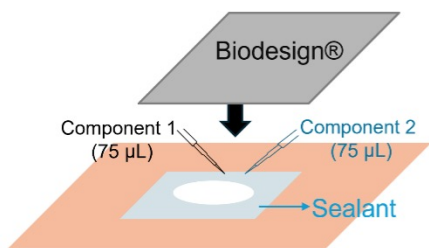

**Top application**

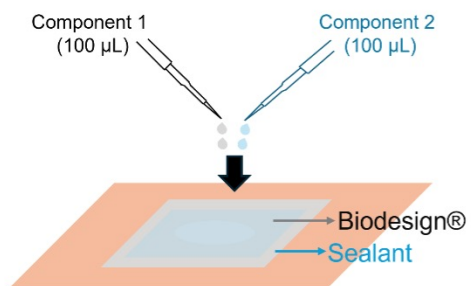

(b)

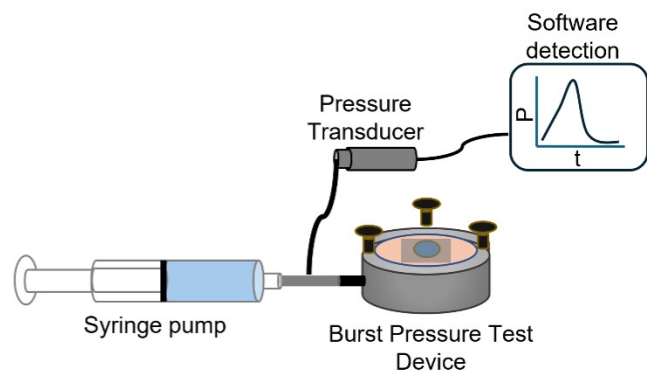

Supplement: Supplementary file 2 — Supporting File 2: alr70132‐sup‐0002‐Figure.pdf [file ALR-16-418-s003.pdf]
